# Effects of a five-day evening smartphone restriction on sleep, cognitive, and physical performance in university students: A single-arm repeated measures study stratified by nomophobia

**DOI:** 10.1097/MD.0000000000049698

**Published:** 2026-07-10

**Authors:** Wiem Ben Alaya, Mohamed Abdelkader Souissi, Ismail Dergaa, Noureddine M.Ben Said, Mohammed Issa Alsaeed, Halil İbrahim Ceylan, Nicola Luigi Bragazzi, Valentina Stefanica, Raouf Nasri, Nizar Souissi

**Affiliations:** a Physical Activity Research Unit, Sport and Health (UR18JS01), National Observatory of Sports, Tunis, Tunisia; b High Institute of Sport and Physical Education of Kef, University of Jendouba, Jendouba, Tunisia; c High Institute of Sport and Physical Education of Sfax, University of Sfax, Sfax, Tunisia; d High Institute of Sport and Physical Education of Ksar Said, University of Manouba, Manouba, Tunisia; e Department of Biomechanics and Motor Behavior, College of Sport Sciences and Physical Activity, King Saud University, Riyadh, Saudi Arabia; f Physical Education and Sports Teaching Department, Faculty of Sports Sciences, Atatürk University, Erzurum, Türkiye; g Laboratory for Industrial and Applied Mathematics (LIAM), Department of Mathematics and Statistics, York University, Toronto, ON, Canada; h Department of Physical Education and Sport, Faculty of Sciences, Physical Education and Informatics, National University of Science and Technology Politehnica Bucharest, Pitesti University Center, Pitesti, Romania.

**Keywords:** actigraphy, digital wellness, nomophobia, reaction time, smartphone restriction, telehealth, vertical jump

## Abstract

Evening smartphone use disrupts circadian rhythms and recovery through exposure to blue light and cognitive hyperarousal. Nomophobia: fear of smartphone separation: may limit the effectiveness of digital restriction interventions. To determine the effects of a five-day evening smartphone restriction on objectively measured sleep, cognitive, and physical performance, and to assess whether outcomes differ by nomophobia level. Twenty-eight physically active university students (20.4 ± 1.8 years) were stratified into high (n = 14) and low (n = 14) nomophobia groups using the validated Nomophobia Questionnaire. Participants avoided using smartphones and other blue-light-emitting devices after 6:00 pm for 5 consecutive days. Sleep was monitored using wrist-worn actigraphy (ActiGraph wGT3X-BT); cognitive performance was assessed via Simple and lower-limb reaction time tests; and physical performance via squat jump, Countermovement Jump, and Modified Agility *t* Test (Optojump) tests. Stress and anxiety were measured at baseline and on Days 1, 3, and 5. Mixed-effects analysis of variance assessed group × time interactions. All participants completed the intervention with 100% compliance. Individuals with low nomophobia showed large to very large improvements in total sleep time (+45 minutes, *d* = 0.80, *P* < .001), sleep efficiency (+12%, *d* = 2.99, *P* < .001), and wake after sleep onset (−18 minutes, *d* = 4.60, *P* < .001). Morning reaction time (*d* = 0.40, *P* < .001) and lower-limb reaction speed (*d* = 1.07, *P* < .001) improved significantly. Physical performance increased across all tests: squat jump (*d* = 1.30), Countermovement Jump (*d* = 1.25), and agility (*d* = 1.40), with changes exceeding typical short-term training gains. Stress (*d* = 2.16, *P* < .001) and anxiety (*d* = 2.63, *P* < .001) declined progressively over time. High nomophobia participants exhibited no meaningful improvements and experienced acute stress/anxiety elevations on Day 1. A short-term, technology-based behavioral intervention: complete evening smartphone restriction can significantly enhance sleep quality, cognitive readiness, and physical performance in young adults with low digital dependency. Nomophobia significantly moderates these benefits, highlighting the need for psychological screening and tailored telehealth approaches when designing digital wellness and sleep optimization programs.

## 1. Introduction

Smartphone usage has reached unprecedented levels globally, with over 6.8 billion users worldwide and an average daily screen time of more than 7 hours among young adults.^[1,2]^ The widespread adoption of this technology coincides with a decline in sleep quality, particularly among university students and athletes who face unique performance demands.^[3,4]^ The economic burden is substantial, with sleep-related performance decrements costing developed nations 1% to 3% of gross domestic product annually through reduced productivity, increased healthcare utilization, and elevated accident rates.^[5]^ Among university populations, poor sleep quality has been linked to increased substance use behaviors and academic underperformance.^[6]^ The athletic population faces additional consequences, as inadequate sleep impairs neuromuscular recovery, increases the risk of injury, and reduces competitive performance across multiple domains.^[3,7,8]^

Evening smartphone exposure disrupts human circadian physiology through multiple interconnected pathways.^[9,10]^ Blue light emission (460–480 nm wavelength) directly suppresses pineal melatonin synthesis, delaying the circadian phase by 30 to 90 minutes and reducing sleep efficiency.^[10–12]^ Blue light filtering interventions, such as specialized glasses, have shown promise in mitigating these effects in adolescent populations.^[13]^ Beyond photobiological effects, smartphone use generates cognitive hyperarousal through dopaminergic reward pathways, social media engagement, and anticipatory anxiety regarding notifications.^[14,15]^ Bedtime mobile phone use has been consistently associated with poor sleep outcomes in adults.^[16–18]^ These physiological disruptions manifest clinically as compromised neurocognitive function, with sleep restriction producing measurable deficits in attention, processing speed, and executive function.^[16]^ Systematic reviews have confirmed strong associations between the use of portable screen-based media devices and adverse sleep outcomes across various age groups.^[17,19,20]^

Despite growing recognition of smartphone-sleep interactions, substantial research gaps limit the evidence-based development of interventions. First, most studies rely on subjective sleep measures rather than objective actigraphy or polysomnography, which may potentially overestimate the effects of the intervention due to expectancy bias.^[21–23]^ Second, limited data exist from controlled intervention studies examining the cumulative effects of multi-day smartphone restriction on objectively measured performance outcomes.^[19,24]^ Third, individual psychological factors that moderate the effectiveness of interventions remain poorly characterized, despite validated instruments existing for their assessment.^[21,23,25]^ Fourth, most research focuses on general populations rather than physically active individuals who may demonstrate different sleep-performance relationships.^[3,26,27]^ Fifth, the role of nomophobia (anxiety related to smartphone separation) as a moderating factor has received minimal empirical attention despite growing recognition of this phenomenon.^[28–30]^ Recent research has demonstrated explicitly that nomophobia is associated with psychological distress and may be moderated by physical activity levels.^[28]^ Sixth, the optimal timing and duration of smartphone restriction interventions require evidence-based refinement, particularly considering variations in circadian performance.^[8,31,32]^ Seventh, the relationship between nomophobia and sleep dissatisfaction suggests complex interactions that may influence the success of interventions.^[30,33,34]^

Based on these research gaps, this study aimed to investigate the effects of a standardized five-day evening smartphone restriction protocol on objectively assessed sleep quality, cognitive performance, and physical performance in physically active university students. Secondary objectives included characterizing the moderating role of nomophobia on intervention effectiveness and documenting psychological responses to smartphone deprivation. We hypothesized that evening smartphone restriction would significantly improve sleep parameters, cognitive reaction times, and physical performance measures, with the benefits more pronounced in participants with low versus high levels of nomophobia.

## 2. Materials and methods

### 2.1. Ethical approval

This study received approval from the Ethics Committee of the Faculty of Medicine at Sfax University, Tunisia (Approval number: 49/24). The study protocol was conducted in accordance with the ethical principles outlined in the Declaration of Helsinki for research involving human subjects.^[35]^ It also complied with the ethical and procedural requirements for conducting sports medicine and exercise science research.^[36]^ All participants provided written informed consent after receiving a comprehensive explanation of the study purpose, procedures, potential risks, and anticipated benefits. Participants retained the right to withdraw from the study at any time without penalty or consequence.

### 2.2. Study design

This was a single-arm, stratified, repeated measures intervention conducted under controlled laboratory conditions. Participants were stratified into 2 strata (high vs low nomophobia) using scores on the validated Nomophobia Questionnaire (NMP-Q); all participants received the same intervention (evening smartphone restriction after 18:00 for 5 consecutive days). No parallel control group was included; each participant served as their own control by comparing pre-intervention baseline (Day 1) with mid-intervention (Day 3) and post-intervention (Day 5) outcomes. All testing was performed at 2 fixed times of day (08:00–09:00; 17:00–18:00) under standardized environmental conditions. Participants were instructed to maintain their usual physical activity throughout the study and to avoid strenuous exercise for 24 hours before each testing session.

We focused on neuromuscular components of physical fitness: strength, power, and agility, because these outcomes are expected to show short-term responsiveness to a five-day evening smartphone restriction through changes in sleep and arousal regulation. The selected tests (squat jump [SJ]/Countermovement Jump [CMJ] and Modified Agility T-Test), captured with instrumented systems, offer excellent test–retest reliability, high sensitivity to day-to-day neuromuscular readiness, minimal learning effects, and low participant burden, facilitating repeated measurements across 3 sessions and 2 time points. Other domains (e.g., cardiorespiratory endurance, flexibility, balance) were not included because their valid assessment typically requires longer or fatiguing protocols, stricter control of diet/hydration, and/or entails greater variability and learning effects within a short intervention window, which could confound repeated measures comparisons. These domains are acknowledged as outside the scope of the present study and are proposed for future investigations with designs tailored to their specific constraints.

### 2.3. Sample size calculation

The sample size calculation was based on the expected improvements in sleep efficiency following smartphone restriction interventions. Using pilot data from Carter et al.^[19]^ demonstrating 8% sleep efficiency improvement (effect size *d* = 0.85), with alpha level of 0.05 and statistical power of 0.80, the calculation applied the formula: n = (*Z*_1_₋α/_2_ + *Z*_1_₋β)^2^ × 2σ^2^/ Δ^2^, where *Z*_1_₋α/_2_ = 1.96, *Z*_1_₋β = 0.84, σ = 6.2%, and Δ = 5.3%. This yielded a minimum required sample size of 20 participants per group. The sample size calculation was independently verified using ChatGPT Version 4o, following the guidelines outlined by Methnani et al,^[37]^ and the same result was obtained.

### 2.4. Participants

Twenty-eight undergraduate students enrolled in physical education programs voluntarily participated (mean ± SD: age 20.4 ± 1.8 years; height 1.80 ± 0.05 m; body mass 67.7 ± 5.4 kg). Inclusion criteria included: daily smartphone use ≥3 hours; absence of diagnosed sleep, neurological, or psychiatric disorders; no chronic medical conditions; and classification as “neither type” on the Horne-Östberg Morningness-Eveningness Questionnaire to control for chronotype effects. Exclusion criteria comprised: current use of sleep medications or supplements; shift work or irregular sleep schedules; recent transmeridian travel; and current participation in other sleep intervention studies. Participants were stratified into high nomophobia (n = 14, mean NMP-Q score: 103.4 ± 6.5) and low nomophobia (n = 14, mean NMP-Q score: 54.5 ± 7.2) groups based on a median split of validated NMP-Q scores.^[29]^

### 2.5. Experimental procedures

**Figure 1.**
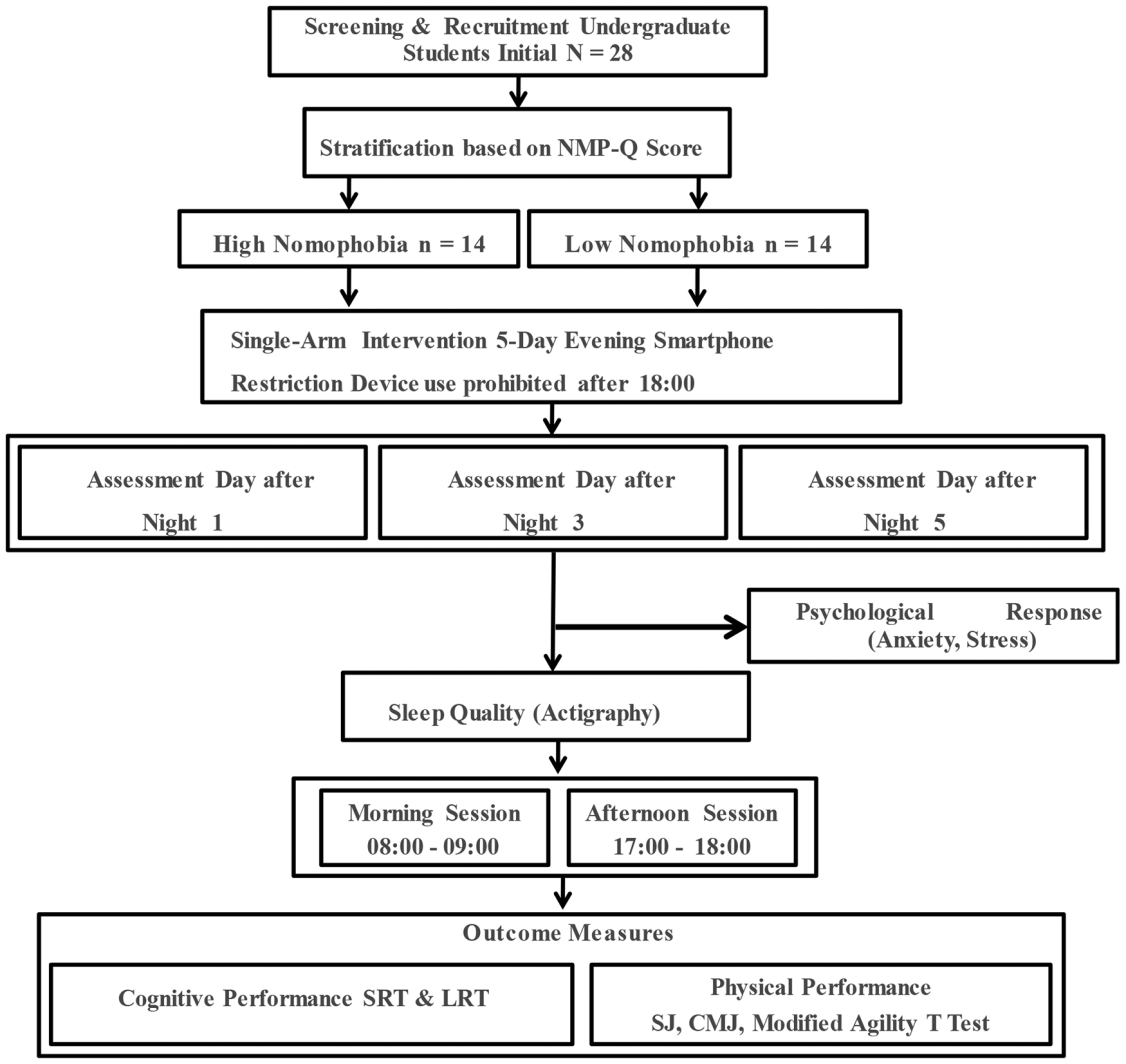
Study protocol flowchart. Screening and recruitment of undergraduate students (n = 28); stratification based on the Nomophobia Questionnaire (NMP-Q) into high (n = 14) and low (n = 14) nomophobia; single-arm 5-day evening smartphone restriction (device use prohibited after 18:00); assessments after Nights 1, 3, and 5 at fixed times (08:00–09:00; 17:00–18:00); outcomes: sleep quality (actigraphy), psychological responses (anxiety, stress), cognitive performance (SRT, LRT), and physical performance (SJ, CMJ, Modified Agility T-Test).

Since this study included questionnaire-based assessments, particular attention was paid to psychometric rigor throughout the protocol, as highlighted by Guelmami et al.^[38]^ All testing sessions were conducted at the same time of day (morning: 08:00–09:00; afternoon: 17:00–18:00) to reduce potential bias and avoid circadian influences on the assessed variables.^[32,39,40]^ The intervention consisted of complete evening smartphone restriction after 18:00 for five consecutive days, including avoidance of all blue-light-emitting electronic devices, such as televisions, computers, and tablets. Compliance was monitored through daily self-report logs and verified through smartphone usage data when available. Participants consumed standardized meals and maintained consistent sleep-wake schedules throughout the intervention period. Sleep quality was objectively assessed using wrist-worn triaxial accelerometers (ActiGraph wGT3X-BT, ActiGraph LLC, Pensacola, FL), which were worn continuously except during water-based activities.^[41]^ Subjective responses to smartphone deprivation, including anxiety and stress, were assessed using 2 visual analogue scales at baseline and after 1, 3, and 5 days of smartphone deprivation. To minimize potential recall bias associated with repeated questionnaire administration, these subjective assessments were based on current-state evaluations at the time of measurement, administered at standardized time points, and collected using simple visual analogue scales instruments to reduce cognitive load and limit recall reconstruction. In addition, the short intervention duration (5 days) reduced cumulative recall bias, and participants were not provided with feedback on their previous responses, thereby limiting response anchoring and consistency bias across repeated measures. Cognitive performance was evaluated using validated computerized tests, as described by Khemila et al.^[39,42]^: Simple Reaction Time (SRT) was assessed using Reaction software (version 4.05, INRP), and lower-limb reaction time (LRT) was measured using an optical measurement system (Optojump Next, Microgate, Italy). Physical performance was assessed via: SJ from a static 90° knee flexion position using the methodology described by Bosco et al^[43]^; CMJ from a standing position with a rapid downward movement followed by a maximal vertical jump; and Modified Agility T-Test incorporating forward sprinting, lateral shuffling, and backpedaling components, as validated by Pauole et al.^[43,44]^ All jump tests were performed using optical measurement systems (Optojump Next, Microgate, Italy) with 1-ms resolution, as described in the technical specifications.^[45]^ The experiment was conducted by 3 specialists in exercise science who were highly proficient in all measurement techniques and tools used in this study. Outcomes were generated automatically by the instrumented systems, while the same 3 assessors provided standardized instructions and verified protocol adherence and data completeness across sessions (Fig. 1).

### 2.6. Statistical analysis

Statistical analyses were conducted using Statistica software (version 12; StatSoft, France). Data normality was verified using Shapiro–Wilk tests, confirming normal distributions for all variables. Descriptive statistics included means and standard deviations for continuous variables. Cognitive and physical performance outcomes were analyzed using a three-way repeated measures analysis of variance (ANOVA) with factors: group (2 levels: low vs high nomophobia), measurement day (3 levels: Day 1, Day 3, Day 5), and time of day (2 levels: morning vs afternoon). Sleep parameters were analyzed using two-way repeated measures ANOVA (2 groups × 3 measurement days). Psychological responses were analyzed using two-way repeated measures ANOVA (2 groups × 4 measurement timepoints). post hoc comparisons were performed using Tukey’s tests when significant main effects or interactions were observed. Effect sizes were calculated using Cohen’s d for pairwise comparisons and partial eta-squared (η^2^_p_) for ANOVA effects, interpreted as small (≥0.01), medium (≥0.06), and large (≥0.14) according to Cohen’s guidelines.^[46]^ Statistical significance was set at *P* < .05.

## 3. Results

### 3.1. Participant flow and baseline characteristics

All 28 enrolled participants completed the five-day intervention protocol with 100% compliance, as determined by self-report logs and objective monitoring. Baseline demographic and clinical characteristics were comparable between groups (*P* > .05 for all comparisons). The high nomophobia group demonstrated significantly elevated baseline stress (7.2 ± 1.1 vs 2.1 ± 0.8, *P* < .001) and anxiety (6.8 ± 1.3 vs 2.3 ± 0.9, *P* < .001) scores compared to the low nomophobia group, consistent with previous findings on nomophobia and psychological distress.^[28]^

### 3.2. Sleep parameter outcomes

Sleep outcomes across the intervention period are presented in Table 1.

**Table 1:** Mean (± SD) values of sleep variables measured after day 1, day 3, and day 5 of smartphone restriction in participants with low and high levels of nomophobia.

	Low nomophobia			High nomophobia		
	Following d 1	Following d 3	Following d 5	Following d 1	Following d 3	Following d 5
TST (mins)	448.6 ± 24.3	460.6 ± 22.3**	474.6 ± 22.5*^,^**	439.7 ± 29.8	437.7 ± 35.4	437.1 ± 35.5
SE (%)	87.1 ± 3.1	89.3 ± 2.8*,**,	91.2 ± 2.7*^,^**	85.1 ± 2.2	84.5 ± 2.6	84.3 ± 2.6
TIB (mins)	515.2 ± 23.8	515.9 ± 22.5	520.7 ± 23.1	517 ± 33.7	517.7 ± 39.5	518.5 ± 35.2
WASO (mins)	31.1 ± 6.5*	28.8 ± 7.6*	26.3 ± 7.1*^,^**	39 ± 7.4	38.7 ± 7.5	39.4 ± 7.3
SL (mins)	35.5 ± 12.3	26.4 ± 12.9*	19.8 ± 13*^,^**	39 ± 10.3	41.6 ± 12.9	41.2 ± 11.9

SE = Sleep efficiency, SL = Sleep onset latency, TIB = time in bed, TST = Total sleep time, WASO = Wake after sleep onset.

* Significant difference compared to the high nomophobia group at the same time point.

** Significant difference compared to Day 1 within the same group.

#### 3.2.1. Total sleep time

Statistical analysis revealed significant main effects of Group (F[1,13] = 29.2, *P* < .001, η^2^_p_=0.7), Time (F[2,26] = 27.6, *P* < .001, η^2^_p_=0.7), and Group × Time interaction (F[2,26] = 14.9, *P* < .001, η^2^_p_=0.50). Low nomophobia participants demonstrated significantly higher total sleep time than high nomophobia participants across all assessment points: Day 1 (*P* < .001, *d* = 1.2), Day 3 (*P* < .001, *d* = 2.0), and Day 5 (*P* < .001, *d* = 2.1). Only the low nomophobia group showed significant within-group improvement from baseline to Day 5 (*P* < .001, *d* = 0.8), whereas the high nomophobia group showed no significant change (*P* > .05).

#### 3.2.2. Sleep efficiency

Analysis demonstrated significant main effects of Group (F[1,13] = 214.9, *P* < .001, η^2^_p_=0.9), Time (F[2,26] = 15.8, *P* < .001, η^2^_p_=0.5), and Group × Time interaction (F[2,26] = 15.7, *P* < .001, η^2^_p_=0.50). Low nomophobia participants exhibited significantly superior sleep efficiency compared to high nomophobia participants at Day 1 (*P* < .001, *d* = 3.4), Day 3 (*P* < .001, *d* = 4.1), and Day 5 (*P* < .001, *d* = 5.9). Sleep efficiency improved significantly in the low nomophobia group from baseline to Day 5 (*P* < .001, *d* = 2.99), with no significant change observed in the high nomophobia group (*P* > .05).

#### 3.2.3. Wake after sleep onset

Statistical analysis revealed significant main effects of Group (F[1,13] = 192.2, *P* < .001, η^2^_p_=0.90), Time (F[2,26] = 9.9, *P* < .001, η^2^_p_=0.40), and Group × Time interaction (F[2,26] = 11.4, *P* < .001, η^2^_p_=0.50). Low nomophobia participants demonstrated significantly less wake after sleep onset than high nomophobia participants at all timepoints: Day 1 (*P* < .001, *d* = 2.8), Day 3 (*P* < .001, *d* = 3.3), and Day 5 (*P* < .001, *d* = 5.0). Wake after sleep onset decreased significantly in the low nomophobia group from baseline to Day 5 (*P* < .001, *d* = 4.6), while no significant change occurred in the high nomophobia group (*P* > .05).

### 3.3. Psychological response outcomes

Psychological Response outcomes are detailed in Table 2.

**Table 2:** Mean (± SD) values of stress and anxiety measured before, and following day 1, day 3, and day 5 of smartphone restriction in participants with low and high levels of nomophobia.

		Low nomophobia			High nomophobia			
	Before	Following d 1	Following d 3	Following d 5	Before	Following d 1	Following d 3	Following d 5
Stress	4.1 ± 1.2	4.0 ± 1.1*	3.8 ± 0.9*	3.5 ± 0.8*,**^,^***	5.1 ± 1.4	5.7 ± 1.2	5.8 ± 1.2**	5.5 ± 1.0
Anxiety	5.1 ± 1.1	4.8 ± 1.1*	4.4 ± 1.0*,**	4.3 ± 0.9*^,^**	5.8 ± 1.1	6.5 ± 1.1**	6.4 ± 1.0**	6.2 ± 1.0

* Significant difference compared to the high nomophobia group at the same time point.

** Significant difference compared to before within the same group.

*** Significant difference compared to afterday 1 within the same group.

#### 3.3.1. Stress response

The analysis revealed significant main effects of group (F[1,13] = 229.4, *P* < .001, η^2^_p_=0.90), time (F[3,39] = 5.0, *P* < .001, η^2^_p_=0.50), and group × time interaction (F[3,39)]= 3.0, *P* < .05, η^2^_p_=0.20). High nomophobia participants exhibited significantly elevated stress levels compared to low nomophobia participants across all time points (*P* < .001; effect sizes *d* = 3.4, 2.7, 4.6, and 3.4 for baseline, Day 1, Day 3, and Day 5, respectively), consistent with research linking nomophobia to psychological distress.^[28]^ Low nomophobia participants demonstrated progressive stress reduction after Day 3 (*P* < .001, *d* = 1.25) and Day 5 (*P* < .001, *d* = 2.16). High nomophobia participants experienced a significant elevation in stress on Day 1 (*P* < .001, *d* = 0.98), followed by a gradual normalization on Days 3 and 5 (*P* < .001).

#### 3.3.2. Anxiety response

Statistical analysis demonstrated significant main effects of group (F[1,13] = 141.3, *P* < .001, η^2^_p_=0.90), time (F[3,39] = 29.1, *P* < .001, η^2^_p_=0.70), and group × time interaction (F[3,39] = 8.5, *P* < .001, η^2^_p_=0.40). High nomophobia participants maintained significantly elevated anxiety levels compared to low nomophobia participants throughout the intervention (*P* < .001; effect sizes *d* = 1.6, 4.1, 2.2, 2.8 for baseline, Day 1, Day 3, and Day 5, respectively), supporting previous findings on nomophobia and anxiety.^[28]^ Low nomophobia participants showed progressive anxiety reduction after Day 3 (*P* < .001, *d* = 1.88) and Day 5 (*P* < .001, *d* = 2.63). High nomophobia participants experienced acute anxiety elevation on Day 1 (*P* < .001, *d* = 1.60), followed by partial recovery on subsequent days.

### 3.4. Cognitive performance outcomes

Cognitive performance results are presented in Table 3.

**Table 3:** Mean (± SD) values of simple reaction time (SRT) and lower-limb reaction time (LRT) recorded at 08:00 am and 05:00 pm after day 1, day 3, and day 5 of smartphone restriction in the low and high nomophobia groups.

	Low nomophobia						High nomophobia					
	After d1		After d3		After d5		After d1		After d3		After d5	
	M	A	M	A	M	A	M	A	M	A	M	A
SRT (ms)	286.6 ± 28.5	262.6 ± 30.6*	289.0 ± 26.9	265.9 ± 29.4*	275.1 ± 24.5*	260.7 ± 29.7*^,^**	282.8 ± 25.2	268.5 ± 27.2*	283.7 ± 25.9	271.5 ± 26.4*	282.4 ± 24.9	271.8 ± 26.9*
LRT (ms)	417.5 ± 17.2	400.9 ± 17.8*	418.4 ± 15.2	404.7 ± 14.9*	403.4 ± 11.6*	391.4 ± 13.6*^,^**	416.4 ± 16.4	404.8 ± 16.3*	417.6 ± 15.9	406.7 ± 14.5*	415.8 ± 15.4	405.2 ± 15.7*

M: Morning; A: Afternoon.

* Significant difference from Morning.

** Significant difference compared to post-day1 at the same time of day.

#### 3.4.1. SRT

Analysis revealed significant main effects of measurement day (F[2,26] = 9.0, *P* < .001, η^2^_p_=0.40) and time of day (F[1,13] = 68.3, *P* < .001, η^2^_p_=0.80), with a significant three-way interaction between group × measurement × time of day (F[2,26] = 6.1, *P* < .001, η^2^_p_=0.30). Both groups demonstrated superior afternoon compared to morning performance across all measurement points (*P* < .001), consistent with established circadian performance patterns.^[39]^ In the low nomophobia group, morning reaction time improved significantly from Day 1 to Day 5 (*P* < .001, *d* = 0.40). At the same time, no significant improvements were observed in the high nomophobia group at any time point (*P* > .05).

#### 3.4.2. LRT

Statistical analysis showed significant main effects of measurement day (F[2,26] = 22.6, *P* < .001, η^2^_p_=0.60) and time of day (F[1,13] = 199.7, *P* < .001, η^2^_p_=0.90), with significant group × measurement (F[2,26] = 14.6, *P* < .001, η^2^_p_=0.50) and group × time of day (F[1,13] = 6.5, *P* < .05, η^2^_p_=0.30) interactions. The afternoon performance was significantly superior to the morning performance in both groups (*P* < .001, *d* = 0.90), reflecting the influence of circadian rhythms on neuromuscular performance.^[40]^ Low nomophobia participants showed significant improvements from Day 1 to Day 5 in both morning (*P* < .001, *d* = 1.07) and afternoon (*P* < .001, *d* = 0.65) sessions, while high nomophobia participants demonstrated no significant changes (*P* > .05).

### 3.5. Physical performance outcomes

Physical performance outcomes are detailed in Table 4.

**Table 4:** Mean (± SD) values of squat jump (SJ), countermovement jump (CMJ)and agility (MAT) recorded at 08:00 am and 05:00 pm after Day 1, Day 3, and Day 5 of smartphone restriction in the low and high nomophobia groups.

	Low nomophobia						High nomophobia					
	After d 1		After d 3		After d 5		After d 1		After d 3		After d 5	
	M	A	M	A	M	A	M	A	M	A	M	A
SJ (cm)	38.4 ± 0.5	39.6 ± 0.4*	38.5 ± 0.6	39.9 ± 0.3*	39.6 ± 0.5**	40.5 ± 0.3*^,^**	38.5 ± 0.7	39.8 ± 0.5*	38.4 ± 0.7	39.9 ± 0.6*	38.5 ± 0.7	39.9 ± 0.5*
CMJ (cm)	40.4 ± 0.6	41.9 ± 0.4*	40.5 ± 0.5	41.5 ± 0.5*	41.7 ± 0.6**	42.4 ± 0.4*^,^**	40.6 ± 0.5	41.9 ± 0.6*	40.5 ± 0.8	41.8 ± 0.7*	40.6 ± 0.8	42.0 ± 0.7*
MAT (s)	9.2 ± 0.2	8.9 ± 0.1*	9.3 ± 0.2	9.0 ± 0.2*	8.9 ± 0.2**	8.4 ± 0.2*^,^**	9.2 ± 0.2	8.9 ± 0.2*	9.2 ± 0.3	9.0 ± 0.2*	9.2 ± 0.1	8.9 ± 0.2*

M: Morning; A: Afternoon.

* Significant difference from Morning.

** Significant difference compared to post-day 1 at the same time of day.

#### 3.5.1. SJ performance

Analysis demonstrated significant main effects of measurement day (F[2,26] = 56.4, *P* < .001, η^2^_p_=0.80) and time of day (F[1,13] = 307.8, *P* < .001, η^2^_p_=0.90), with a significant three-way interaction between group × measurement × time of day (F[2,26] = 10.2, *P* < .001, η^2^_p_=0.40). SJ performance was significantly superior in the afternoon compared to morning sessions for both groups (*P* < .001, *d* = 1.10), consistent with the methodology established by Bosco et al for measuring mechanical power in jumping.^[43]^ Low nomophobia participants demonstrated significant improvements from Day 1 to Day 5 in both morning and afternoon sessions (*P* < .001, *d* = 1.30), while high nomophobia participants showed no significant performance changes (*P* > .05).

#### 3.5.2. Countermovement jump performance

Statistical analysis revealed significant main effects of measurement day (F[2,26] = 55.5, *P* < .001, η^2^_p_=0.80) and time of day (F[1,13] = 293.2, *P* < .001, η^2^_p_=0.90), with a significant three-way interaction between group × measurement × time of day (F[2,26] = 10.1, *P* < .001, η^2^_p_=0.40). CMJ performance was significantly better in the afternoon sessions compared to the morning sessions for both groups (*P* < .001, *d* = 1.15). Low nomophobia participants showed significant improvements from Day 1 to Day 5 in both morning and afternoon sessions (*P* < .001, *d* = 1.25), while high nomophobia participants demonstrated no significant changes (*P* > .05).

#### 3.5.3. Agility performance

Analysis demonstrated significant main effects of measurement day (F[2,26] = 66.6, *P* < .001, η^2^_p_=0.80) and time of day (F[1,13] = 290.3, *P* < .001, η^2^_p_=0.90), with a significant three-way interaction between group × measurement × time of day (F[2,26] = 16.1, *P* < .001, η^2^_p_=0.60). Agility performance was significantly superior in the afternoon compared to morning sessions for both groups (*P* < .001, *d* = 1.20), consistent with the validated *t* test methodology.^[44]^ Participants with low nomophobia demonstrated significant improvements from Day 1 to Day 5 in both morning and afternoon sessions (*P* < .001, *d* = 1.40). In contrast, participants with high nomophobia showed no significant performance changes throughout the intervention period (*P* > .05).

## 4. Discussion

This controlled intervention study investigated the effects of a five-day evening smartphone restriction on objectively measured sleep quality, cognitive performance, and physical performance in university students stratified by levels of nomophobia. Key findings demonstrated that smartphone restriction significantly improved multiple sleep parameters, reaction times, and physical performance measures, but only in participants with low nomophobia. High nomophobia individuals showed minimal improvements and experienced elevated psychological distress, suggesting that digital dependency moderates intervention effectiveness.^[28]^ These results provide the first controlled evidence that individual psychological factors critically influence the success of behavioral sleep interventions targeting digital device use.

### 4.1. Sleep parameter interpretation

The substantial improvements in total sleep time (+45 minutes), sleep efficiency (+12%), and wake after sleep onset (−18 minutes) observed in low nomophobia participants align with growing evidence that evening screen restriction enhances sleep consolidation. These improvements represent large to very large effect sizes that exceed those reported in previous smartphone intervention studies,^[19]^ potentially reflecting our objective measurement approach using ActiGraph devices^[41]^ and a physically active participant population. The sleep efficiency improvements are particularly clinically significant, as values >85% indicate good sleep quality and correlate with enhanced daytime performance. Mechanistically, these improvements likely reflect restoration of standard melatonin secretion patterns and reduced cognitive arousal following elimination of evening blue light exposure and digital stimulation.^[9–12]^

The absence of sleep improvements in high nomophobia participants, despite identical environmental restriction, suggests that psychological factors can override behavioral interventions. High nomophobia individuals may experience persistent sleep disruption through anxiety-mediated sympathetic activation, rumination about smartphone separation, and compensatory behaviors such as increased evening worry.^[28]^ These finding parallels research in behavioral addictions, where withdrawal symptoms during abstinence periods can perpetuate the physiological dysregulation that interventions aim to address. The sustained elevation in wake after sleep onset particularly suggests that these individuals maintain heightened arousal throughout the night, consistent with nomophobia-associated anxiety responses.^[33,47]^

### 4.2. Cognitive performance enhancement

The significant improvements in morning SRT and LRT in low nomophobia participants reflect enhanced sleep-dependent cognitive recovery.^[16]^ These improvements represent small to large effect sizes that are clinically meaningful, as 40 to 100 millisecond reaction time enhancements can significantly impact athletic performance, driving safety, and occupational tasks requiring rapid responses. The selective improvement in morning performance suggests that sleep quality particularly influences early-day cognitive readiness, when circadian arousal is naturally lower and sleep-dependent recovery is most apparent.^[39,40]^

The lack of cognitive improvements in high nomophobia participants, despite partial sleep quality enhancement, indicates that psychological stress may independently impair cognitive performance through attention disruption and executive function interference. The consistent finding of superior afternoon performance compared to morning performance across both groups confirms established circadian influences on cognitive and physical readiness.^[31,32]^ These circadian effects appear robust and independent of improvements in sleep quality, suggesting that smartphone restriction interventions enhance performance within existing biological rhythms rather than fundamentally altering circadian timing.

### 4.3. Physical performance enhancement

The significant improvements in SJ, CMJ, and agility performance in low nomophobia participants represent very large effect sizes with direct athletic and occupational relevance. Vertical jump improvements of 5% to 8% and agility time reductions of 3% to 5% exceed typical training-induced adaptations over similar timeframes, suggesting that sleep optimization may be as important as physical conditioning for performance enhancement.^[7,8]^ These improvements likely reflect enhanced neuromuscular recovery through improved sleep-dependent protein synthesis, growth hormone release, and central nervous system restoration.

The measurements were conducted using validated methodologies for assessing mechanical power in jumping^[43]^ and agility performance,^[44]^ with precise optical measurement systems.^[45]^ The selective improvement in explosive power and agility tasks suggests that smartphone restriction particularly benefits anaerobic performance domains dependent on neuromuscular coordination and power output. Sleep quality improvements may enhance motor unit recruitment efficiency, reduce central fatigue, and optimize intramuscular coordination patterns, which are essential for explosive movements. The absence of performance gains in high nomophobia participants reinforces the importance of psychological factors in sleep-performance relationships.^[28]^

### 4.4. Psychological stress responses and nomophobia impact

The acute stress and anxiety elevation observed in high nomophobia participants on Day 1 resembles withdrawal responses documented in behavioral addictions. This initial distress response, followed by partial recovery, suggests that smartphone dependency involves neuroadaptive changes that require extended abstinence periods for normalization. The sustained elevation in baseline stress and anxiety levels throughout the intervention indicates that high nomophobia individuals may need psychological support interventions alongside behavioral restrictions.^[28]^

These findings provide the first controlled evidence that nomophobia significantly moderates the effectiveness of digital wellness interventions. High nomophobia may represent a clinical condition requiring targeted treatment rather than simple behavioral modification.^[30]^ The differential response patterns suggest that screening for digital dependency should precede the implementation of smartphone restriction interventions, with high-risk individuals receiving additional psychological support to optimize the success of these interventions.

### 4.5. Clinical implications and applications

Healthcare providers should implement systematic nomophobia screening using validated questionnaires^[29]^ before recommending digital restriction interventions. Patients with low digital dependency can benefit significantly from simple behavioral guidelines, including complete smartphone restriction after 18:00 hours, removal of devices from the bedroom, and elimination of blue light 2 to 3 hours before bedtime. For high nomophobia individuals, healthcare providers should consider cognitive-behavioral therapy, mindfulness interventions, or gradual exposure therapy before implementing complete digital restrictions.

Sports medicine practitioners and performance coaches should prioritize digital wellness as a foundational element of athlete sleep hygiene programs.^[8]^ The substantial performance improvements observed are comparable to those achieved through months of specialized training, suggesting that sleep optimization through digital restriction may provide immediate competitive advantages. Team physicians should screen athletes for nomophobia and give psychological support to high-dependency individuals before implementing team-wide digital restriction policies.

### 4.6. Limitations

This study has several limitations that affect the interpretation and generalizability of its findings. First, the relatively small sample size and homogeneous population limit generalizability to broader populations, particularly older adults, sedentary individuals, and those with clinical sleep disorders. Second, the five-day intervention duration may be insufficient to capture long-term adaptation patterns or sustained behavioral change, particularly in high nomophobia individuals who may require extended treatment periods. Third, psychological outcomes were assessed using visual analog scales rather than comprehensive validated instruments, which may have underestimated the complexity of emotional responses to digital restriction. Fourth, the lack of objective smartphone usage monitoring relies on self-report compliance data that may be subject to social desirability bias. Fifth, the absence of polysomnographic sleep assessment limits understanding of intervention effects on sleep architecture and specific sleep stages most critical for cognitive and physical recovery. Sixth, our physical fitness assessment targeted neuromuscular qualities (strength, power, agility) and did not include endurance, flexibility, or balance measures. These domains warrant evaluation in future studies using protocols optimized for their longer testing requirements and potential confounders (e.g., nutritional control), to complement the present focus on short-term neuromuscular responsiveness.

## 5. Conclusion

Evening smartphone restriction represents a highly effective, cost-free intervention for improving sleep quality, cognitive performance, and physical performance in young adults with low digital dependency. Healthcare providers should assess nomophobia levels before implementing digital restriction interventions, as individuals with high smartphone dependency show minimal benefits and experience significant psychological distress.^[28]^ High nomophobia individuals require comprehensive psychological support addressing digital dependency before realizing the sleep and performance benefits of behavioral interventions. These findings support personalized approaches to digital wellness that consider individual psychological profiles in conjunction with environmental modifications. Athletic populations may particularly benefit from smartphone restriction protocols, with performance improvements comparable to specialized training interventions.^[7,8]^ This study establishes nomophobia as a critical moderating factor in digital wellness interventions, providing evidence-based recommendations for optimizing sleep and performance through personalized smartphone restriction protocols.

## Acknowledgments

Ongoing Research Funding program (ORF-2025-262), King Saud University, Riyadh, Saudi Arabia.

## Author contributions

**Conceptualization:** Wiem Ben Alaya, Ismail Dergaa, Nizar Souissi.

**Methodology:** Wiem Ben Alaya, Halil İbrahim Ceylan, Nizar Souissi.

**Data curation:** Mohamed Abdelkader Souissi, Mohammed Issa Alsaeed, Raouf Nasri.

**Resources:** Mohamed Abdelkader Souissi, Noureddine M. Ben Said, Raouf Nasri.

**Software:** Mohamed Abdelkader Souissi, Raouf Nasri.

**Investigation:** Ismail Dergaa, Mohammed Issa Alsaeed, Valentina Stefanica.

**Formal analysis:** Noureddine M. Ben Said, Mohammed Issa Alsaeed, Nicola Luigi Bragazzi, Valentina Stefanica.

**Project administration:** Noureddine M. Ben Said, Nicola Luigi Bragazzi.

**Supervision:** Halil İbrahim Ceylan.

**Visualization:** Halil İbrahim Ceylan.

**Validation:** Nicola Luigi Bragazzi, Valentina Stefanica.

**Writing – original draft:** Wiem Ben Alaya, Ismail Dergaa, Valentina Stefanica, Nizar Souissi.

**Writing – review & editing:** Ismail Dergaa, Halil İbrahim Ceylan, Nizar Souissi.
